# Efficiency of Tragacanth gum coating enriched with two different essential oils for deceleration of enzymatic browning and senescence of button mushroom (*Agaricus bisporus*)

**DOI:** 10.1002/fsn3.1000

**Published:** 2019-03-25

**Authors:** Mahshid Nasiri, Mohsen Barzegar, Mohammad Ali Sahari, Mehrdad Niakousari

**Affiliations:** ^1^ Faculty of Agriculture, Department of Food Science and Technology Tarbiat Modares University Tehran Iran; ^2^ Faculty of Agriculture, Department of Food Science and Technology Shiraz University Shiraz Iran

**Keywords:** button mushroom, natural coating, *Satureja khuzistanica*, shelf life, *Zataria multiflora* Boiss

## Abstract

The effect of Tragacanth gum (T) coating containing (100, 500, and 1,000 mg/L) *Satureja khuzistanica* essential oil (S), *Zataria multiflora* Boiss. essential oil (Z), and (1,000 mg/L) sodium metabisulfite (M) on mushroom (*Agaricus bisporus*) enzymatic browning and postharvest quality was examined throughout 16 days of cold storage. Mushroom respiration rate, soluble solids content (SSC), percentage of open caps, and sensory quality as well as factors related to browning such polyphenol oxidase (PPO), phenylalanine ammonia lyase (PAL), and peroxidase (POD) activities were figured out. The significant decrease in respiration rate, cap opening delay, and SSC enhancement was observed after treating mushrooms with TZ and TS. Moreover, TZ‐ and TS‐treated mushrooms prevented enzymatic browning through inhibiting PPO and POD activities and increasing activity of PAL over the storing term. Additionally, the influence of TZ5 and TS5 (containing 500 mg/L essential oil) coatings was validated by sensory evaluation through protecting the overall quality of button mushrooms over the storage. Thus, Tragacanth coating enriched with essential oils might be an encouraging nomination for improving the modality of button mushroom and expanding its shelf life.

## 1 INTRODUCTION

*Agaricus bisporus* Imbach (white button mushroom), with the first reports on the cultivation date from 1707, from France, produces about 32% of total edible mushroom in the world, extensively recognized for its nutritional, medicinal, and organoleptic attributes (Gao, Feng, & Jiang, 2014; Khan et al., 2014; Rzymski, Mleczek, Niedzielski, Siwulskid, & Asecka, 2017). *A. bisporus* is a perishable food product that its shelf life may be limited by bacterial spoilage or enzymatic browning, depending on handling and storage conditions (Sapers, Miller, Pilizota, & Kamp, 2001).

A substitutable way for postponing ripening and alleviating postharvest decay of fruit is achieved by application of natural products (Tripathi & Dubey, 2004). Edible coatings can provide an additional protective layer for fresh products and can also give the same effect as modified atmosphere storage in amending internal gas composition (Park, 1999). Application of semi‐permeable obstacle versus O_2_, CO_2_, moisture, and solute movement has been revealed to expand the postharvest life of fruit and vegetables, increase the water barrier, inhibit microbial contamination, maintain flavor, reduce the degree of shrinkage distortion, and retard fat oxidation (Ali, Maqbool, Ramachandran, & Alderson, 2010; Jiang, 2013; Jiang, Feng, Zheng, & Li, 2013). Polysaccharides, proteins, and lipids are different sources for production of edible films and coatings with particular characteristics (Raeisi, Tajik, Aliakbarlu, Mirhosseini, & Hosseini, 2015). Fruit quality protection has been achieved using several edible coatings, such as chitosan in fresh‐cut strawberry (Campaniello, Bevilacqua, Sinigaglia, & Corbo, 2008), gum Cordia in Chilgoza (Haq, Alam, & Hasnain, 2013), and gum Arabic in mango fruit (Khaliq, Mohamed, Ali, Ding, & Ghazalic, 2015).

Tragacanth gum (T) is one of the three major exudate gums, which possesses a unique range of functionalities (Verbeken, Dierckx, & Dewettinck, 2003). This natural and acidic polysaccharide secretes spontaneously or with a scrape on the different species of *Astragalus* plant. This gum composed of 30%–40% Tragacanthin (water‐soluble and extremely branched) and 60%–70% bassorin (insoluble but water‐swellable fraction)*.* Forms and structures of these two constituents are different. The ratio of Tragacanthin and bassorin strongly depends on variety (Azarikia & Abbasi, 2016).

Safety aspects of chemical additives caused increasing interest of application of natural preservative coatings with antimicrobial agents (Jiang, Feng, & Zheng, 2012), such as gum Arabic, aloe vera, chitosan containing thyme essential oil (Bill, Sivakumar, Korsten, & Thompson, 2014), chitosan coating augmented with thyme oil (Jiang et al., 2012), and carboxymethyl cellulose‐based coatings containing *Zataria multiflora* Boiss. essential oil and grape seed extract (Raeisi et al., 2015). These researches have revealed that essential oils (EOs) and extracts of various herbs and spices could be used as natural food preservers due to their antimicrobial acting. Like other biopolymers, Tragacanth gum is known as an appropriate carrier for natural antimicrobial and antioxidant complexes (Taban, Rahimi, Saharkhiz, Hadian, & Zomorodian, 2013).

*Zataria multiflora* Boiss (Labiatae) is an herbal plant with several thin, hard, and highly ramified stems from family which found naturally in Iran, Pakistan, and Afghanistan. This plant with traditional title of Avishan Shirazi (in Iran) has been used as antiseptic, anesthetic, antispasmodic, and flavor composition in broad range of foods in Iran. The essential oil of *Z. multiflora* Boiss. contains compounds with important pharmaceutical, antimicrobial, and antioxidant effects (Fathi, Sahari, Zangiabadi, & Barzegar, 2011; Kordsardouei, Barzegar, Sahari, & Ebrahimipour, 2015). Phenolic compounds, such as carvacrol and thymol, are the major components of the *Z. multiflora Boiss*. essential oil (Z) (Dashipour et al., 2015).

Another plant that is separated through southern part of Iran including Ilam, Lorestan, and Khuzestan provinces is *Satureja khuzistanica* Jamzad (Lamiaceae). It is a small shrub with branched stem approximately 30 cm high, densely leafy, covered with short white hairs (Moazeni, Saharkhiz, Hoseini, & Mootabi Alavi, 2012). Analgesic and an antiseptic characteristic of plant are considered by the people in southern region of Iran. A main component of wild *S. khuzistanica* (≤90%) is monoterpene carvacrol (Hashemi, Niakousari, Saharkhiz, & Eskandari, 2012).

The purpose of this study was to investigate the effect of Tragacanth gum coating, individually and/or in combination with *Z. multiflora Boiss*. essential oil (S) and *S. khuzistanica* essential oil (Z) and sodium metabisulfite (M) as a chemical preservative (which widely used in the mushroom processing industry), on mushroom tissue browning, some physicochemical, and as well as sensorial changes during 16 days of storage in refrigerator (4 ± 1°C).

## 2 MATERIALS AND METHODS

### 2.1 Plant material

Button mushrooms were gathered from a local farm in Shiraz, Iran. Mushrooms were selected from the similar flower and zone of the shed so as to reduce probable alteration affected by cultivation and environment. During one hour of harvesting, the mushrooms were conveyed to the test center and then kept in dimness at 4 ± 1°C and 90% relative humidity (RH). Mushrooms having good physical shape without any damage were selected to be used in the experiments.

### 2.2 *Zataria multiflora* Boiss. and *Satureja khuzistanica* essential oils

Two essential oils (EOs) were acquired from Giah Essans (Gorgan, Iran). Their characteristics sheets indicate 98% pureness.

### 2.3 Tragacanth gum and coating treatments

The best kind of gum was obtained from a local shop, washed, dried, milled, and determined the optimum concentration of gum solution (0.6%), and ten different treatments were prepared based on Nasiri, Barzegar, Sahari, & Niakousari, 2017: (a) control (C, water washed, without coating); (b) Tragacanth gum coating (T); (c) gum coatings containing 100 mg/L S (TS1), (d) 500 mg/L S (TS5), (e) 1,000 mg/L S (TS10); (f) gum coatings containing 100 mg/L Z (TZ1), (g) 500 mg/L Z (TZ5), (h) 1,000 mg/L Z (TZ10); (i) 1,000 mg/L sodium metabisulfite (M); and (j) gum coating containing 1,000 mg/L metabisulfite (TM). They were placed in polyethylene (HDPE) containers and were kept for 16 days at 4 ± 1°C and 95% RH pending further analysis, and every 4 days, three replicates from each treatment group were analyzed.

### 2.4 Respiration rate

Respiration rate of samples was measured using method described by Li, Zhang, and Yu (2006).

### 2.5 Soluble solids content

Mushrooms were crushed in a pounder. The juice was pressed and analyzed for the soluble solids content (SSC). The measurement was carried out in a CARL Zeiss refractometer model 78969 (Germany) at 25°C.

### 2.6 Percent open caps and overall acceptability

The principles of evaluating the percentage of open caps were in accordance with development of the umbrella‐like form of the cap followed by detachment of veil. The percent open caps were measured using known number of mushrooms based on Equation (1):

(1) $$\%\;\text{Open}\;\text{caps}=\frac{N_{\text{oc}}}{N_{t}}\times 100$$

where *N*_oc_ = number of opened cap mushrooms; *N*_t_ = total number of mushrooms.

The consumer acceptance of the product was studied using 10 panelists with age ranging from 25 to 40 years. Panelists were trained based on the method described by Ares, Parentelli, Gámbaro, Lareo, and Lema (2006). Mushrooms were served in closed, odorless plastic containers at room temperature. After opening polyethylene bags, evaluations were performed within 2 hr in order to avoid loss of off‐odors. The overall acceptability based on color, texture, and percent opened caps were done by round‐table basis using four‐point scale where 1 = poor, 2 = fair, 3 = good, and 4 = excellent (Jiang et al., 2011).

### 2.7 Determination of protein content and PPO, PAL, and POD activities

Enzymatic activity determined as explained by Gao et al. (2014). The protein content determination was carried out as the method of Bradford (1976), with application of bovine serum albumin as a standard.

Polyphenol oxidase (EC 1.10.3.2) activity was investigated using incubating 0.5 ml of enzyme extract in 2.5 ml of buffered substrate (100 mM sodium phosphate, pH = 6.4, and 50 mM catechol), then detecting the change of absorbance at 398 nm. The quantity of enzyme caused 0.01 increases in absorbance per minute under the specified conditions defines one unit of PPO activity. The specific PPO activity was represented as U/mg protein.

Phenylalanine ammonia lyase (EC 4.3.1.5) measured spectrophotometrically using the substrate trans‐cinnamic acid at 290 nm. Three ml of the reaction mixture, containing 0.8 ml supernatant and 50 mM l‐phenylalanine in sodium borate buffer (200 mM, pH = 8.8), was incubated at 37°C; after 90 min, the reaction was interrupted by ice water. The amount of enzyme caused 0.01 absorbance rise in 1 hr under the assay conditions defined as one unit of PAL activity. The specific PAL activity was expressed as U/mg protein.

Peroxidase (EC 1.11.1.7) activity was analyzed by determining the absorbance of guaiacol. 50 mM sodium phosphate buffer (pH = 6.0), 5 mM guaiacol, 5 mM H_2_O_2_, and 50 μl of tissue extract constructed the reaction mixture. One unit of POD activity was distinguished as the quantity of enzyme that leads to change of 0.01 absorbance per minute at 470 nm under the specified conditions. The specific POD activity was defined as U/mg protein.

### 2.8 Statistical analysis

Experiments were conducted in triplicates. Data were analyzed using SPSS (version 16; IBM, USA). Analysis of variance (ANOVA) followed by Duncan's multiple range test was used to distinguish the treatments at *p* < 0.05.

## 3 RESULTS AND DISCUSSION

### 3.1 Effect of gum coating containing EOs on respiration rate

**Figure 1:**
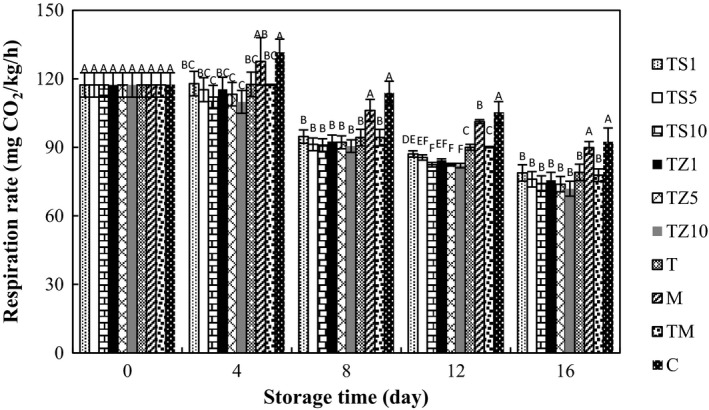
Effect of essential oil‐incorporated gum coating on respiration rate of button mushrooms stored at 4°C for 16 days. Vertical bars represent standard errors of means. C: control (without coating); M: 1,000 mg/L sodium metabisulfite; T: coating with Tragacanth gum (TG); TM: TG coating containing 1,000 mg/L metabisulfite; TS1: TG coating containing 100 mg/L of *Satureja khuzistanica* essential oil (S); TS5: 500 mg/L S; TS10: 1,000 mg/L S; TZ1: TG coating containing 100 mg/L *Zataria multiflora* Boiss. essential oil (Z); TZ5: 500 mg/L Z; TZ10: 1,000 mg/L Z

Figure 1 demonstrated the respiration rates of button mushrooms over 16 days of storage. The primary respiratory rate defined as CO_2_ accumulation of mushrooms at first day was around 117.33 mg CO_2_ kg^−1^ hr^−1^. During storage, significant reduction in respiration rate was found, which could be attributed to mushroom deterioration (*p* < 0.05). At the end of storage time, the respiration rates of the control (92.4 mg CO_2_ kg^−1^ hr^−1^) and M (84.8 mg CO_2_ kg^−1^ hr^−1^) samples were higher than TZ1 (75.6 mg CO_2_ kg^−1^ hr^−1)^, TZ5 (73.9 mg CO_2_ kg^−1^ hr^−1^), TZ10 (71.9 mg CO_2_ kg^−1^ hr^−1^), TS1 (78.8 mg CO_2_ kg^−1^ hr^−1^), TS5 (76.0 mg CO_2_ kg^−1^ hr^−1^), TS10 (74.2 mg CO_2_ kg^−1^ hr^−1^), TM (77.8 mg CO_2_ kg^−1^ hr^−1^), and T (79.0 mg CO_2_ kg^−1^ hr^−1^) samples. Samples treated with TZ10 displayed a considerably inferior respiration rate than other treated or untreated samples. The respiration rate of mushrooms treated with gum contained EOs did not reveal substantial variation. The process by which cells obtain chemical energy by the consumption of oxygen and the release of carbon dioxide is known as respiration (Xu, Tian, Mab, Liu, & Zhang, 2016). Earlier investigation indicated that essential oil treatments had the effective role in falling the respiration rate of nectarine fruits (Abd El Wahab, 2015), Tabarzeh grape (Abdolahi, Hassani, Ghosta, Bernousi, & Meshkatalsadat, 2010), and Crimson seedless grape (Abd El Wahab, Abd El Wahab, & Kamel, 2014). Furthermore, respiration rate reduction was observed in apple piece coated by alginate (Wong, Tillin, Hudson, & Pavlath, 1994) and mushrooms treated with alginate coating (Jiang, 2013). Internal gas atmosphere modification, affected by TG coating, reduced CO_2_ production and respiration rate. The permselectivity, gas obstacle specifications, and affiliation with relative humidity temperature of the edible coating applied to surface will appearance a crucial role in the variations in interior O_2_ and CO_2_ levels. Obviously, the extreme limitation of gas exchange can cause anaerobiosis and the development of off‐flavor (Jiang et al., 2012). Contrariwise, scientists have discovered that the essential oils isolated from Satureja could display antifungal specification (Farzaneh, Kiani, Sharifi, Reisi, & Hadian, 2015; Taban et al., 2013) and the antimicrobial effects of ZEO applied in different coatings such as chitosan (Moradi, Tajik, Razavi Rohani, & Oromiehie, 2011), carboxy methyl cellulose (Dashipour et al., 2015), and zein (Moradi, Tajik, Razavi Rohani, & Mahmoudian, 2016) have been reported. Consequently, interaction of gum and EOs could decrease respiration rate without increment of off‐flavor.

### 3.2 Effect of gum coating containing essential oils on SSC

**Figure 2:**
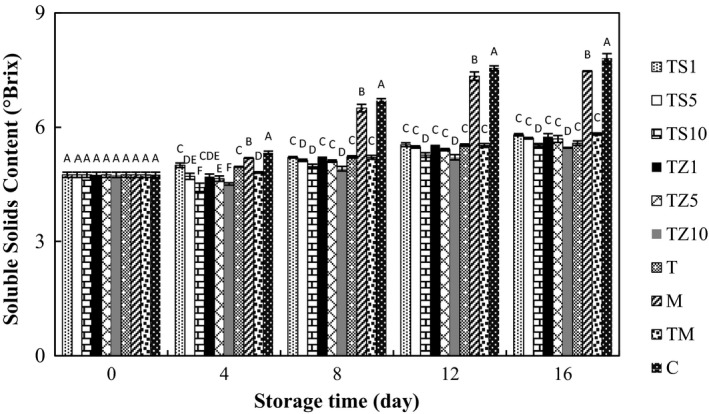
Effect of essential oil‐incorporated TG coating on soluble solids content of button mushrooms stored at 4°C for 16 days. Vertical bars represent standard errors of means. For abbreviations see Figure 1

Soluble solids content enhancement was gradual during storage time (Figure 2). The control and MS samples exposed the highest SSC; it extended 7.80 and 7.47°Brix, compared with 5.80, 5.71, 5.53, 5.75, 5.69, 5.46, 5.82, and 5.58°Brix in TZ1‐, TZ5‐, TZ10‐, TS1‐, TS5‐, TS10‐, TM, and gum‐coated mushrooms (T), respectively. The observed surge in SSC is a sign of high respiration rate and retarding the senescence process (Jafri, Jha, Bunkar, & Ram, 2013). Another factor contributed to SSC increase in aged mushroom might be the cell wall polysaccharides and hemicellulose solubilization (Jiang et al., 2012). The increase in SSC of TZ10 and TS10 samples was slower and more gradual than the other samples. The coatings provided a promising semi‐permeable film on the surface of the fruit, adjusting the interior atmosphere by decreasing O_2_ and/or enhancing CO_2_ and suppressing production of ethylene. The synthesis and use of metabolites consequences lower SSC, due to the slower hydrolysis of carbohydrates to sugars, decelerated by decreased respiration rates (Ali et al., 2010; Rohani, Zaipun, & Norhayati, 1997). The same effects were found by Khaliq et al. (2015) presented a slow increase in SSC concerning mango coated with Arabic gum.

### 3.3 Effect of gum coating containing essential oils on percentage of open caps and overall acceptability

After 16‐day storage, the proportion of opened caps mushrooms increased in all samples (Table 1). Control (C) and M‐treated samples had higher opened caps mushrooms, 82.2% and 80.0%, respectively. Over the same period, the percentage of opened caps mushrooms coated with TZs, TSs, and T were in the range 66.7%–75.6%. According to Jiang (2013), mushroom cap opening leads to increased water loss, which in turn leads to mushroom dryness. Cohesive forces of water and other hydrophilic molecules, such as proteins responsible for the well position of the caps and veil in mushrooms, diminished over the storage, as a result of water loss increment. TG coating alone and in combination with each of the essential oils attenuated water loss, the cap opening of mushrooms was lower, and the least changes (66.7%) were related to mushrooms coated with TZ10.

**Table 1:** Effect of Tragacanth gum coating containing two essential oils on percentage of open caps of samples

Treatment	Time (day)			
	4	8	12	16
TS1	11.7 ± 4.4^Ad^	36.4 ± 3.4^ABCDc^	57.0 ± 6.3^ABCb^	75.4 ± 2.4^CDa^
TS5	11.4 ± 4.2^Ad^	29.7 ± 5.2^CDc^	53.3 ± 0.0^Cb^	70.5 ± 1.0^EFa^
TS10	11.1 ± 3.8^Ad^	29.4 ± 6.9^CDc^	52.2 ± 1.9^Cb^	68.3 ± 2.8^Fa^
TZ1	11.6 ± 8.5^Ad^	31.9 ± 4.7^BCDc^	54.6 ± 2.2^BCb^	73.0 ± 2.0^DEa^
TZ5	11.3 ± 3.6^Ad^	29.5 ± 3.4^CDac^	52.4 ± 5.3^Cb^	68.4 ± 1.7^Fa^
TZ10	10.7 ± 8.1^Ad^	28.9 ± 3.8^Dc^	51.1 ± 3.8^Cb^	66.7 ± 0.7^Fa^
T	13.3 ± 0.0^Ad^	37.8 ± 3.9^ABc^	57.8 ± 3.9^ABCb^	75.6 ± 2.2^CDa^
M	15.6 ± 7.7^Ad^	40.0 ± 0.0^Bc^	62.2 ± 3.9^ABb^	80.0 ± 1.3^ABa^
TM	11.9 ± 4.1^Ad^	37.1 ± 2.5^ABCc^	58.3 ± 5.6^ABCb^	77.0 ± 1.6^BCa^
C	17.8 ± 3.6^Ad^	42.2 ± 3.9^Ac^	64.5 ± 3.9^Ab^	82.2 ± 3.9^Aa^

^a^Mean of three replications ± standard deviation. Means in the same row with different small letters are significantly different (*p* < 0.05). Means in the same column with different capital letters are significantly different (*p* < 0.05). C: control (without coating); M: 1,000 mg/L sodium metabisulfite; T: coating with Tragacanth gum; TM: TG coating containing 1,000 mg/L metabisulfite; TS1: TG coating containing 100 mg/L of *Satureja khuzistanica* essential oil (S); TS5: 500 mg/L S; TS10: 1,000 mg/L S; TZ1: TG coating containing 100 mg/L *Zataria multiflora* Boiss. essential oil (Z); TZ5: 500 mg/L Z; TZ10: 1,000 mg/L Z.

Overall acceptability (based on color, texture, and percentage samples opened caps) reduced during storage (Table 2). Based on the assessment given by the sensory panel members, the control and MS failed to be acceptable following 16 days of storage. Over the same period of storage, mushrooms coated with Tragacanth gum and essential oils did not display any unsuitable characteristics and received an overall acceptability of about 2.17 from the panelists. These results demonstrate the effectiveness of the TZ5 and TS5 in delaying mushroom appearance deterioration. Jiang et al. (2012) reported the reaction of polyphenol oxidase (PPO) enzyme and the action of bacteria and mold on the mushroom tissue result in browning of mushrooms. As Tragacanth gum and gum containing essential oils coatings could lower the activity of spoilage organisms, such as *Pseudomonas*, undertaking for phenolic compounds oxidation to form brown‐colored melanins, improving the appearance and color.

**Table 2:** Effect of Tragacanth gum coating containing two essential oils on overall acceptability of samples

Treatment	Time (day)			
	4	8	12	16
TS1	3.6 ± 0.1^ABCa^	3.4 ± 0.1^Aab^	2.4 ± 0.1^ABb^	2.1 ± 0.2^Ac^
TS5	4.1 ± 0.1^ABa^	3.5 ± 0.1^Aab^	3.2 ± 1.1^Aab^	2.1 ± 0.1^Ab^
TS10	4.0 ± 0.2^ABCa^	3.4 ± 0.2^Ab^	2.6 ± 0.4^ABc^	2.1 ± 0.1^Ac^
TZ1	3.9 ± 0.0^ABCa^	3.4 ± 0.1^Aab^	2.6 ± 0.6^ABbc^	2.1 ± 0.1^Ac^
TZ5	4.1 ± 0.2^Aa^	3.5 ± 0.2^Aa^	2.7 ± 0.4^ABb^	2.2 ± 0.0^Ac^
TZ10	4.0 ± 0.2^ABCa^	3.4 ± 0.1^Aab^	2.6 ± 0.5^ABbc^	2.1 ± 0.0^Ac^
T	3.9 ± 0.2^ABCa^	3.3 ± 0.2^ABa^	2.3 ± 0.5^ABb^	2.0 ± 0.2^Ab^
M	3.9 ± 0.3^ABCa^	2.9 ± 0.1^Bb^	2.0 ± 0.1^ABc^	1.7 ± 0.1^Bc^
TM	3.5 ± 0.6^BCa^	3.3 ± 0.1^Aa^	2.4 ± 0.4^ABab^	2.1 ± 0.2^Ab^
C	3.5 ± 0.1^Ca^	2.6 ± 0.1^Cb^	1.7 ± 0.2^Bc^	1.3 ± 0.1^Cd^

^a^Mean of three replications ± standard deviation. Means in the same row with different small letters are significantly different (*p* < 0.05). Means in the same column with different capital letters are significantly different (*p* < 0.05). For abbreviations see Table 1.

### 3.4 Effect of gum coating containing EOs on PPO activity

**Figure 3:**
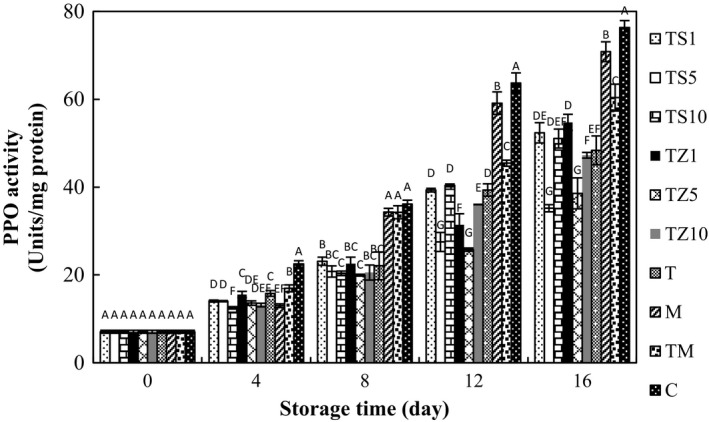
Effect of essential oil‐incorporated TG coating on PPO activities of button mushrooms stored at 4°C for 16 days. Vertical bars represent standard errors of means. For abbreviations see Figure 1

The activity of PPO increased during the storage period. PPO activity of control mushrooms increased swiftly without peaks and was considerably greater than samples coated with TG and TG containing essential oils (Figure 3). TG coatings might be slightly permeable especially to oxygen, thus preventing PPO to access to oxygen. Browning has been studied in other fruits, and discoloration correlated well with PPO activity. The inhibition of the PPO activity by coating fruits and vegetables was reported by numerous investigators: chitosan coating on litchi fruit (Jiang, Li, & Jiang, 2005), carrageenan coating on fresh‐cut banana (Bico, Raposo, Morais, & Morais, 2009), and different edible coatings on fresh‐cut “Royal Gala” apple (Bertrand, Raposo, Morais, & Morais, 2015). In the present study, a high activity of PPO leads to a dark and unpleasant appearance of mushrooms. This result is in a good agreement with those reported by the above researchers. Here, there exist associations among the PPO activity and the color parameters in mushrooms; enhancing the PPO activity leads to the decrease in L* values and the increase in BI, indicating the dependency of enzymatic browning reactions to the PPO. In general, PPO acceleration the monophenols (monophenolase) hydroxylation and the oxidation of *o*‐diphenols into *o*‐quinones (diphenolase). Therefore, in the presence of oxygen, polymerization and creation of the unwanted brown pigments took place (Amin, 2016). In general, treatments with essential oils prompt a decrease in the activity of PPO. The studies conducted by Amin (2016) who sprayed celery, basil, and peppermint essential oils on Anna apple and Gao et al. (2014) in fumigation of button mushroom with clove, cinnamaldehyde, and thyme essential oils show similar trends observed in our studies and those reported by several previous investigators. Compared to the control and M samples, those treated by TG containing essential oils preserved more ascorbic acid content (Data not shown). This may be attributed to the pH lowering action of ascorbic acid particularly at high concentration, which decreases the pH below the optimum value in turn inhibiting the catalytic action of PPO (Sapers, 1993).

### 3.5 Effect of gum coating containing essential oils on POD activity

**Figure 4:**
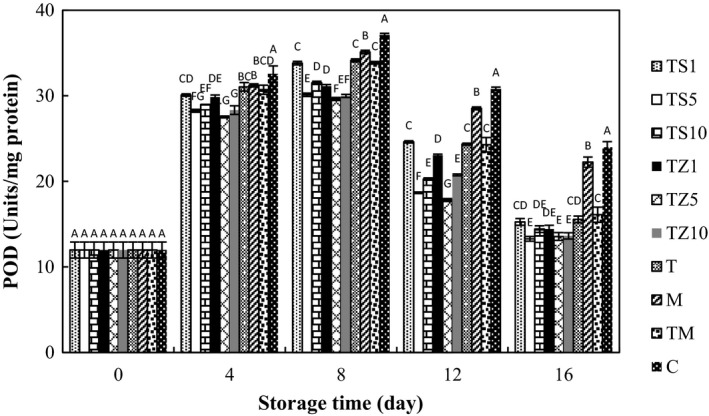
Effect of essential oil‐incorporated TG coating on POD activities of button mushrooms stored at 4°C for 16 days. Vertical bars represent standard errors of means. For abbreviations see Figure 1

In all treatments, pattern of POD activity was similar during 16 days of storage, reaching a peak and then declined. The POD activities in TZ‐ and TS‐coated mushrooms were significantly lower than that in C (Figure 4). Unpleasant quality alterations, such as discoloration, off‐flavor, and nutritional destruction through handling, transportation, packaging, processing, and storage of fruit and vegetables, are consequences of enzymatic browning. These reactions resulted mostly from enzymes such as peroxidase (Mousavizadeh, Sedaghathoor, & Khorami, 2011). The POD association with enzymatic browning and antioxidant defense has been revealed by former investigation (Lin et al., 2017). The increase in peroxidase is a sign of fruit quality deterioration and is relevant to the enzymatic browning process because *o*‐phenols could encourage fruit and vegetable products to be darkened over the processing and preservation and could display reducing function in the enzyme reaction (Amin, 2016). The quality features and mushroom decay may be affected by different enzymatic changes in chemical constituents. Also, POD could affect odor and taste and deteriorate eating and nutritional qualities of mushrooms (Baardseth, 1979; Fasidi & Kadiri, 1991). For that reason, it is required to inactivate the peroxidase enzyme to minimize deterioration.

It is acceptable that edible coatings practice as protective barrier on the surface of fresh product, diminish oxygen supply, slow down ripening and senescence, and, subsequently, postpone browning of fruits and vegetables. Several preservation methods, based on chitosan coating (Eissa, 2007) and alginate coating (Jiang, 2013), restricted activities of POD. In addition, application of essential oils resulted in the decrease in the peroxidase enzyme activity, which may account for the inhibition of the mushroom browning. Alikhani, Sharifani, Azizi, Hemati, and Mousavizadeh. (2009) reported the value of natural essential oils in the decrease in activity of the peroxidase enzyme of fruits and vegetables. Mousavizadeh et al. (2011) demonstrated that antioxidative properties of essential oils can lead to peroxidase activity reduction in white and red cabbage. In this study, gum coating‐impregnated EOs reduced POD activity of samples. Thus, it appears that coating influence on browning inhibition is through extenuating oxygen supply and inhibitory properties on POD activity.

### 3.6 Effect of gum coating containing EOs on PAL

**Figure 5:**
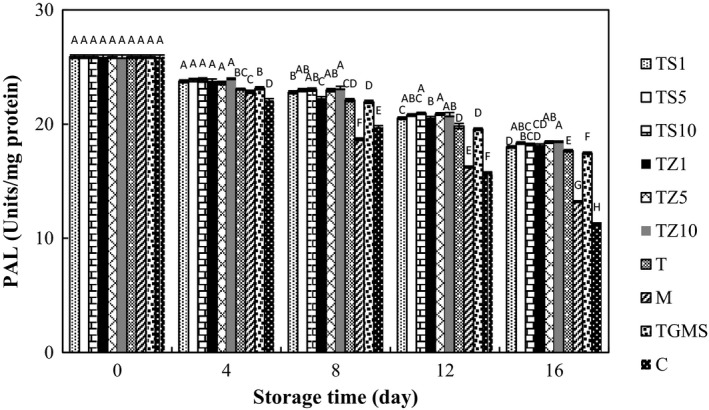
Effect of essential oil‐incorporated TG coating on PAL activities of button mushrooms stored at 4°C for 16 days. Vertical bars represent standard errors of means. For abbreviations see Figure 1

Despite PPO, PAL activities of all samples decreased continuously during storage (Figure 5). PAL is the major enzyme in the biosynthesis of varieties of phenylpropanoid compounds, such as simple phenols, anthocyanin, flavonoid, and lignin (Hiratsuka et al., 2001; Ju, Yuan, Lieu, & Xin, 1995). It is stated that the accumulation of phenols and anthocyanins equated the PAL activity enhancement in some fruits (Wang et al., 2009). Thus, PAL activity was studied in this paper to examine the possible response to TG coating containing EOs. Unlike, Eissa (2007) who reported cationic charge of chitosan caused protein binding and chitosan coating‐treated fresh‐cut mushroom inhibited the increase in PAL activity. Gum coating containing EOs induced increasing in PAL activity compared to control and M samples. It is conceivable that essential oils exhibit positive influence on plant secondary metabolites and motivating biosynthesis of phenolic and anthocyanin compounds by promoting PAL activity (Gao et al., 2014). TZ10, TZ5, TS10, and TS5 were more effective than other treatments in enhancing PAL activity.

## 4 CONCLUSIONS

Inhibition of enzymatic browning is the most important concern in the mushroom's industry. As the food industry tends to decrease the use of chemical preservatives in food products, TG coating contained each essential oils could be considered as a potential source for shelf‐life extension of cold‐stored mushroom. TZ and TS treatments slowed down respiration rate, postponed cap opening, and SSC addition compared with gum‐coated, M‐treated, and control mushrooms. Furthermore, TS5 showed greater influence to inhibit discoloration of button mushrooms. Consequently, these natural products have promising effect to protect the quality and safety of button mushroom. TZ and TS coatings could prevent enzymatic browning through inhibition the activities of PPO and POD, increasing PAL activity during the storage time.

## CONFLICT OF INTEREST

The authors declare that they have no competing interests.

## ETHICAL STATEMENT

Study's protocols and procedures were ethically reviewed and approved by Tarbiat Modares Research Council. Ethics approval and consent to participate are not applicable to this manuscript, since it does not report on or involve the use of any animal or human data or tissues.
